# Using Qualitative System Dynamics Analysis to Promote Inclusive Livestock Value Chains: A Case Study of the South African Broiler Value Chain

**DOI:** 10.3389/fsufs.2021.670756

**Published:** 2021-08-05

**Authors:** Soledad Cuevas Garcia-Dorado, Kevin Queenan, Bhavani Shankar, Barbara Häsler, Tafadzwanashe Mabhaudhi, Gregory Cooper, Rob Slotow

**Affiliations:** 1 SOAS University of London, London, United Kingdom; 2 London School of Hygiene and Tropical Medicine, University of London, London, United Kingdom; 3 Department of Pathobiology and Population Sciences, Royal Veterinary College (RVC) University of London, London, United Kingdom; 4 Department of Geography, The University of Sheffield, Sheffield, United Kingdom; 5 School of Life Sciences, University of KwaZulu-Natal, Durban, South Africa

**Keywords:** small producers, system dynamic analysis, policy and institutional actions, livestock, value chains

## Abstract

Livestock value chains globally are increasingly dualistic, with integrated market-leaders co-existing with comparatively disadvantaged small producers who, nevertheless, support rural livelihoods and food access, and can also contribute to more resilient supply chains. The South African broiler value chain provides a highly illustrative case study. The purpose of this study is to identify potential leverage points for policy intervention to support small and emerging producers in the South African broiler value chain, and to discuss the strengths and limitations of system dynamics approaches to promote inclusive food value chains. This study develops a causal loop diagram (CLD) based on semi-structured stakeholder interviews and policy documents. The main challenges, key variables and causal relationships between them are systematically identified. Variables are coded, generalised and graphically represented, and entry points for intervention and their links to existing policies are mapped. The challenges faced by smallholders in the context of our study can be characterised, using a CLD, as a set of interlinked and reinforcing dynamics which perpetuate existing disadvantages and reinforce duality in the value chain. Key policy entry points have been identified that could be targeted by a coordinated policy package, including: Direct support for infrastructure investment and input access through grants, subsidies or other policies; credit and liquidity provision for day-to-day expenses; creation of aggregation mechanisms for both inputs and outputs; regulations or initiatives that directly target the relationship of farmers with the commercial segment to improve access to day-old-chicks and, finally, training in business and technical skills. Although most of these interventions have been addressed at some point, implementation has been fragmented, failing to fully consider their complementary nature, thus undermining effectiveness. Existing approaches to consensus building and stakeholder participation in system dynamics research can present challenges when it comes to engaging with complex policy processes and issues of conflict of interest that are relevant in the context of smallholder promotion and equitable food systems, but there are promising avenues for addressing. Despite some methodological challenges, we find that there is considerable scope for system dynamics approaches to inform policy for smallholder promotion, even in contexts characterised by complex policy processes.

## Introduction

In recent years, the role of smallholder farmers has increasingly been recognised as crucial to achieving the Sustainable Development Goals (SDG) and to addressing the increasing environmental and socio-demographic pressures on food systems (Abraham and Pingali, 2017).

Although policy focus on smallholder support declined following the structural adjustment policies in the 1980s, many African governments have in recent years renewed their efforts to promote smallholders and local food systems (Dionne and Horowitz, 2016).

In South Africa, despite policy efforts, multiple challenges persist when it comes to the promotion and support of small farmers (von Loeper et al., 2018). The South African broiler value chain, considered a national priority (DAFF, 2014a), offers a highly relevant case study for the analysis of challenges and potential solutions for the promotion of inclusive and equitable livestock value chains.

Complex systems analysis, system dynamics and related approaches, which can be captured under the more general term of “systems thinking”, have been advocated as a new paradigm for the analysis, characterisation and evaluation of food systems (Zhang et al., 2018), agricultural value chains (Orr et al., 2018) and agricultural policy interventions (Douthwaite et al., 2003; Rammelt and Leung, 2017).

Applications cover a diverse range of topics, including the analysis of rural road development (Rammelt and Leung, 2017), water resource management (Winz et al., 2009), urban agricultural planning (Rich et al., 2018), ecosystem-poverty-health interactions (Grace et al., 2017), productivity improvements in local dairy value chains (Lie et al., 2017, 2018)or climate change policy (Totin et al., 2018). Studies in the South African context have applied systems thinking to a number of issues including the future of the South African food system (Pereira, 2014), a characterisation of the South African livestock system (Queenan et al., 2020) or the challenges of smallholders and conservation agriculture (von Loeper et al., 2016).

Systems approaches aim to go beyond siloed analysis of agricultural policies and challenge simplistic representations of agricultural policy impacts, including traditional marketequilibrium research which fails to account for complex mechanisms and reinforcing dynamics (Rammelt and Leung, 2017). Moreover, systems analysis aims to support participation, include diverse types of knowledge and ultimately, and perhaps most importantly for our study, to promote transformative change (Wiek et al., 2012; Mapfumo et al., 2017).

The present study applies causal loop diagrams (CLD), a systems analysis tool, to the issue of smallholder promotion in the South African broiler value chain. Firstly, this research has aimed to identify the challenges faced by smallholders, characterise the dynamic mechanisms underlying them and assess the potential for a transformative policy agenda to promote inclusive value chains (Wiek et al., 2012). Secondly, the challenges of complex systems analysis applied to the issue of smallholder farmer promotion are discussed, providing recommendations for future research in the area.

## Background: The Dualistic Broiler Value Chain In South Africa, Relevance And The Role Of Small And Emerging Producers

In South Africa, small farmers generally belong to historically disadvantaged communities, and their promotion within strategic agricultural sectors is considered a key component of national development strategies (DAFF, 2014a). Improved market access for smallholders is identified as a key element in the National Policy on Food and Nutrition Security (DSD DAFF, 2013) and the integrated maize-soy-poultry value chain, in particular, is a priority sector (DAFF, 2014b). The importance and rapid growth of the poultry sector, particularly broiler meat, is not exclusive to South Africa, however. The emergence of the broiler sub-sector has been identified as a key feature of food system transition in low and middle income countries and as one of the key shifts occurring in global food systems (Enahoro et al., 2018).

In South Africa, the sector is highly industrialised, but dualistic. This dualism, deeply rooted in historical inequalities, is mirrored across other livestock sectors (Queenan et al., 2020) A small number of vertically integrated firms dominate broiler production, providing practically all domestic supply to mainstream, formal channels (Ncube, 2018). The corporate segment is technically competitive in relation to large producers in other major supplying countries, although it compares less favourably in terms of economic efficiency (Davids and Meyer, 2017). Integrated firms co-exist with a large number of medium-small to very small producers who mainly sell live birds or broiler products in the informal market. Supermarkets and fast food retailers are also very powerful players in the industry, and drive production standards to a large extent (Heijden and van der Vink, 2013; DAFF, 2017a; Ncube, 2018).

From a nutrition-security point of view, chicken currently represents the main and most affordable source of animal protein nation-wide (DAFF, 2017a; Jörnling, 2017), and poultry is the largest agricultural sector in terms of value-addition (DAFF, 2017a). Small broiler producers, despite making up a relatively small share of the market, play an important role in supporting rural livelihoods (Louw et al., 2017; Wong et al., 2017). They also provide an affordable source of protein to some of the most vulnerable groups of population in South Africa (Schönfeldt et al., 2013), reaching rural markets and informal settlements that include some of South Africa’s most disadvantaged consumers (DSD DAFF, 2013; Louw et al., 2017). This includes households without fridges or without reliable access to electricity, who therefore prefer to buy live chickens and slaughter them when needed.

Previous literature has described cost structures and challenges faced by small producers in the South African broiler sector, with a focus on assessing their possibilities for inclusion into mainstream value chains (Wynne and Lyne, 2003; Louw et al., 2017). Louw et al. (2017) found limited possibilities for integration of smallholders into mainstream commercial value chains, given the economies of scale involved in broiler production, which imply reduced efficiency for small producers. Despite this disadvantage, smallholders manage to offer affordable prices while making some profit by bypassing supermarkets, which keep a substantial proportion of value added in mainstream value chains, and by supplying niche markets, often selling live birds (Greenberg, 2017; Louw et al., 2017).

Finally, it is worth noting that the challenges faced by small broiler producers affect not only their livelihoods and local food supply, but can also undermine One Health outcomes (Rüegg et al., 2018), such as human health (food safety, zoonoses), animal health (Avian Influenza, antimicrobial use and welfare), and environmental health (adequate waste management). These concerns are not specific to smallholders, but the mechanisms and underlying incentives driving them can differ from those affecting integrated producers, and can show some parallels with challenges experienced by indigenous poultry producers (Malatji et al., 2016).

## Methodology

The methodology used in this study can be characterised as “critical systems thinking,” maintaining “an ethical base to action and choices” and prioritising critical reflection and the inclusion of diverse views throughout the research process (Smith, 2011). This involves adhering to a series of methodological and epistemological principles:

- A “dualistic” philosophy of system dynamics (Han and Kunc, 2015) is adopted, accepting the existence of an objective reality as well as the subjectivity of mental models and seeking pragmatic compromise. This approach, very frequent in current system dynamics practise, departs from earlier, more mechanistic approaches, and is more compatible with social science paradigms (Smith, 2011). The resulting models seek to represent reality, relying on triangulation of different sources of information, while acknowledging and critically examining the influence of participants’ and researchers’ subjective perception of reality.
- Power dynamics and imbalances are considered throughout the research, including in the processes of data collection and interpretation. Critical awareness of power dynamics is used to ensure the inclusion of diverse views, and to guarantee that all participants are provided with an appropriate and safe space to express these views (Smith, 2011).
- Researchers have a duty to consider how their research process and outputs might impact participants and their context, and to aim for “improvement”, understood as “the purposive action of an agent to create change for the better” (Midgley, 2000).

The rest of this section will discuss the study’s approach to stakeholder classification, data collection and analysis.

### Classification of Stakeholders

This study adopted a systems approach, incorporating the perspectives of small producers but also of other actors in the system dealing with smallholders in different roles.

Value chain actors include broiler producers, feed producers, traders/retailers and importers. Additional stakeholders can be broadly categorised into, policy-makers, academic experts, and professionals providing services to producers (such as veterinarians or other professionals providing extension services and training for farmers).

Smallholder farmers are defined, according to the classification provided by the South African Poultry Association (SAPA) as those producing fewer than 40,000 birds per cycle (SAPA, 2016), and include producers housing as little as 100 birds (Louw et al., 2017).

Smallholders or “small producers,” both as used in the literature and as defined by SAPA, refers to a heterogeneous group of actors with differing situations and characteristics (Cousins, 2010). Independent producers within this category, however, are examined together based on the challenges they face related to their exclusion from vertically integrated systems and mainstream value chains. The current approach could be complemented by further analysis of sub-groups of producers or of explicit funds allocation to specific sub-categories. Previous studies have distinguished between medium-small (under 40,000 birds) and very small producers (around 1,000) (Louw et al., 2017). Although there is no formal cut-off, we will refer very small producers or simply small producers as those with an output of under 5,000 birds per cycle, while medium or medium-small producers are above this threshold and closer to the upper limit within the under-40,000 category.

Contract growers are excluded from this category as we consider them to be part of the vertically integrated system. We will interchangeably refer tosmall holders or small and emerging producers. The term emerging farmers is generally used in South Africa to refer to relatively small, independent farmers aiming to grow and become established commercial enterprises (Mabaya et al., 2011). Broiler production, unlike indigenous chicken farming, is generally commercially oriented, even for very small farmers, so we do not talk about “subsistence” farmers as a category.

### Data Collection

Twenty-two (22) semi-structured interviews were carried out with 28 stakeholders in the broiler sector. This study obtained ethics approval from the Royal Veterinary College’s (University of London) Social Science Ethical Review Board (URN SR2018-1624) and through the SOAS (University of London) Ethical Review of Research Projects Procedure (23/April/2019), as well as being covered by the ethics approval granted to the University of Kwa-Zulu Natal, Humanities & Sciences Research Ethics Committee, Protocol number HSS0287/018 for the SHEFS project that this study is part of. Written informed consent was obtained from participants, before interviews were recorded, professionally transcribed verbatim and independently coded by two researchers (SCG-D and KQ). Initial sampling was based on a preliminary systems framework and additional interviewees were contacted by snowballing. Interviews were carried out in Johannesburg (mainly policy makers, SAPA representatives, experts and other national-level actors) and in Kwa-Zulu Natal (small broiler producers, feed producers, traders, providers of training for small farmers and veterinarians and experts). Two interviews were carried out online, using teleconferencing software (Skype).

Participants included representatives of the SA poultry association, the Department of Agriculture Forestry and Fisheries, the Department of Trade and Industry, feed producers, importers, one large contract grower, small informal traders, and professionals who deliver veterinary services and training to small producers. In addition, we interviewed different types of small producers: one medium-small producer, one small registered cooperative run by men, and one small cooperative, run by women (SAPA, 2018). Interview topics included overall perceived trends in the sector and perceived challenges for specific actors and the role of policy in driving the change in the sector and in mediating the identified challenges, as well as the relevant policy actors involved. In addition, questions were asked regarding nutrition security impacts, environmental factors, food safety, and animal health. Although the topics were common across interviews, the order and emphasis on different themes varied to focus on the most relevant issues for each participant. For example, stakeholders involved in exporting or importing were asked specifically about tariffs and import-export regulations, while other stakeholders would only be asked about this if it was identified by them as a relevant driver of changes, challenges or opportunities. Interviews varied in duration, lasting approximately 45 mins and including about 20 questions.

### Policy Document Review

Information from interviews was complemented with a policy document review. The document search and analysis was concurrent with the interview design, data collection and analysis phases. The aim of the document review was to identify policies which can be relevant to the support, livelihoods and performance of smallholder broiler farmers, which were then classified into broad policy areas, including corporate standards, public health, foreign trade, food security and agricultural development. The stated aims of each policy in relation to smallholders were then identified, assessing whether the corresponding policy documents mention smallholder support explicitly as an aim, and whether they make special provisions for smallholders. This analysis was used to frame the study within the existing policy context, increasing its relevance to decision-makers, advocates, and practitioners.

Based on stakeholder interviews, agricultural development policy was identified as the main relevant policy area and is discussed in Section Knowledge, Information and Skills.

### Analysis

The analysis in this study is based on the methodology developed by Kim and Andersen (2012) for the creation of causal loop diagrams based on qualitative transcripts. The main analytical steps are summarised in Table 1. Detailed analysis matrices can be made available as supplementary material or upon request from the authors.

Firstly, we carry out a full thematic analysis of stakeholder interviews and a policy document review. This serves to identify the main challenges faced by small producers in the supply chain and map the policies used to address these challenges.

Secondly, the key segments of the interviews and policy documents are analysed to identify variables and causal relationships between them (Kim and Andersen, 2012). Thirdly, variables are grouped into categories and “generalised” or replaced by more general variables. Finally, variable and causal link tables are translated into a graphical representation and archetypal dynamic structures are identified (Wolstenholme, 2003). The text, tables and diagram are labelled to keep the links between them. Rather than describing every plausible variable within the system, the resulting CLD summarises the key problematic behaviours and causal links emerging from the stakeholder interviews. The resulting CLD is not the only possible formulation. Other researchers, analysing the same information, could focus on different issues and perhaps obtain a different formulation, for example disaggregating different parts of the system. However, the use of strategies to minimise bias through step-by-step model building and triangulation across different sources support the formulation of solid, policy relevant insights and theory.

### Causal Loop Diagrams: Basic Concepts

Based on a full, inductive-deductive thematic analysis of the interviews, a causal loop diagram was developed to serve as a basis for future policy analysis and identification of adequate strategies for value chain improvement. Causal loop diagrams are a systems thinking tool that qualitatively represent variables (nodes) and the relationships between them (links) (Sterman, 2001). A positive (negative) causal link between two nodes is represented by an arrow and implies that a change (increase or decrease) in the corresponding causing variable will lead to a movement of the caused variable in the same (opposite) direction. CLD can represent feedback dynamics, where causes are in turn affected by their outcomes. These feedback loops can be reinforcing (R) or balancing (B). Delays are marked with a double line crossing the corresponding arrow and important feedback loops are named and labelled in the diagrams. In this study, the CLD reflects how participants in the broiler value chain view the system and, in particular, the different challenges faced by smallholders, and the links between them. Distinct dynamics are represented in the form of partial CLD, which are combined into a final CLD.

Interview quotes are marked with an interview-specific code. The analysis of background literature and policy documents, discussed in the following subsection, informs the interpretation and integration of participants’ views of the system into the CLD.

## Findings: Interlinked Challenges For Small-Medium Producers, Dynamic Feedbacks And Causal Loops Formulation

**Figure 1:**
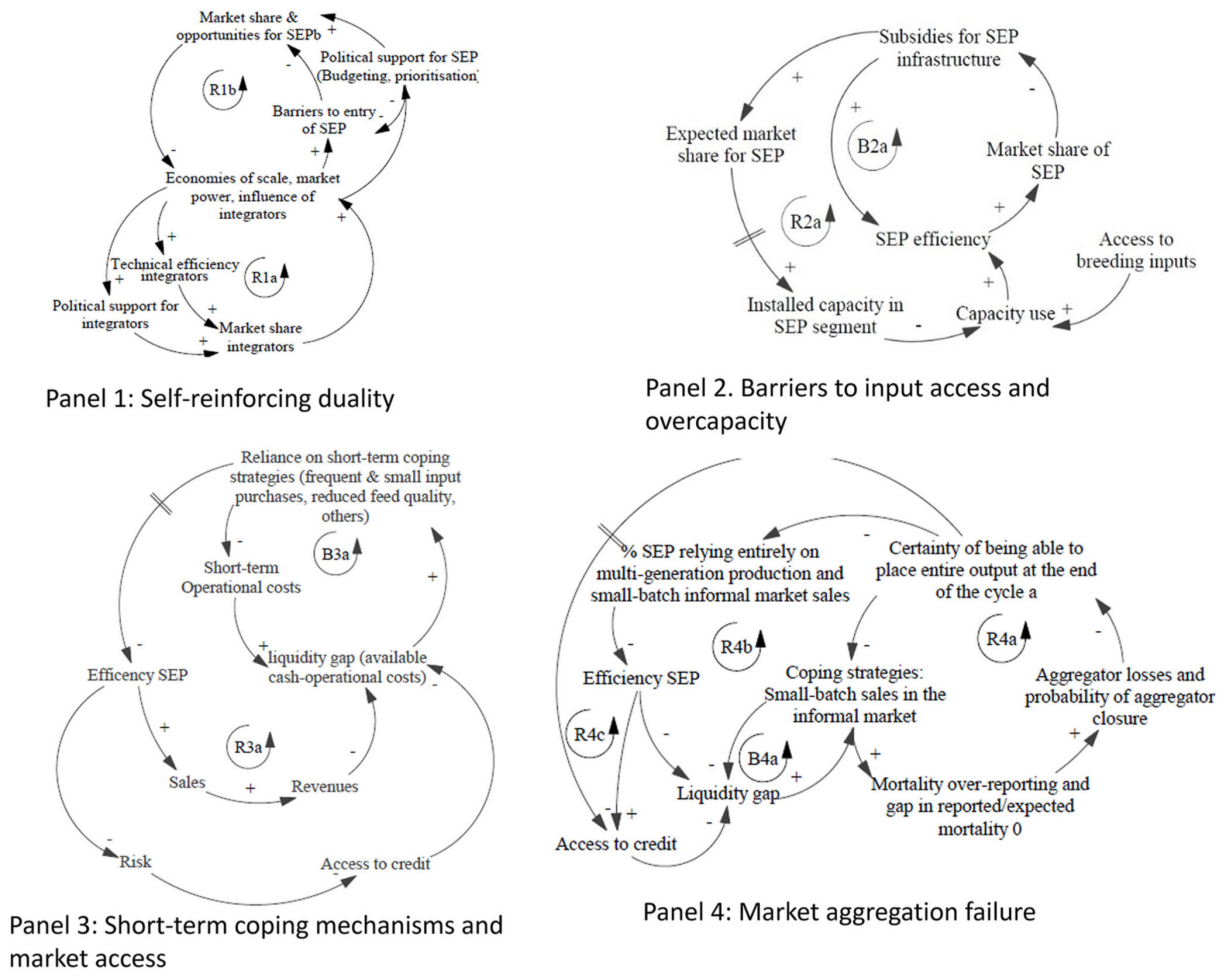
Partial causal loop diagrams. Feedback loops are marked and labelled, R for reinforcing loops and B for Balancing loops. These letters are followed by a number that corresponds to the panel and a letter that differentiates the different feedback loops within one panel (e.g., R1a is the first reinforcing feedback loop in panel 1). Arrows indicate causality. The signs +/- next to an arrow indicate the nature of the relationship between the two variables linked by the arrow. If an increase in the causal variable leads, all else equal, to an increase (decrease) in the caused variable, the sign will be positive (negative). Panels are labelled with short descriptive titles that refer to the main mechanisms described.

**Figure 2:**
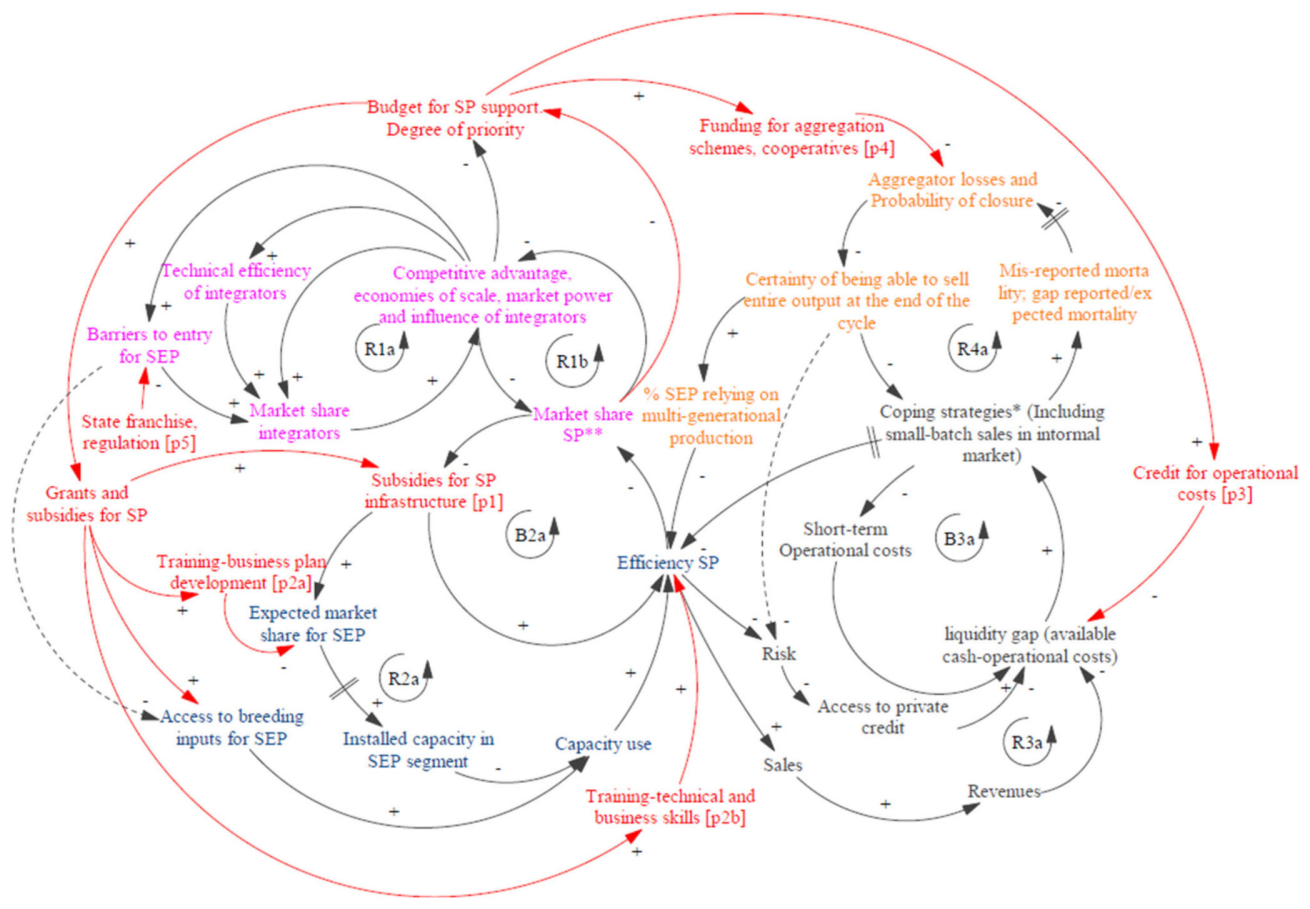
Full causal loop diagram. Feedback loops are marked and labelled, R for reinforcing loops and B for Balancing loops. Arrows indicate causality. The signs +/- next to an arrow indicate the nature of the relationship between the two variables linked by the arrow. If an increase in the causal variable leads, all else equal, to an increase (decrease) in the caused variable, the sign will be positive (negative). SP stands for small producers. Variables corresponding to each Panel in Figure 1 are shown in different colours to improve readability. Pink: Reinforcing duality; blue: barriers to input access and overcapacity; black: short-term coping mechanisms and market access; yellow: failure of market aggregation mechanisms Policy entry points and flows of funds are shown in red. Some lines are dashed to improve readability.

This section presents the findings of this study and the different elements of the causal loop diagram (CLD). Partial diagrams are presented in Figure 1, representing the corresponding dynamics. The full CLD is presented in Figure 2, where the partial diagrams are linked, and policy entry points are shown. Table 2 explains the elements of the full CLD as well as showing the policy entry points and corresponding policies. This table acts both as a summary and extended CLD legend.

### Value Chain Duality as a Self-Reinforcing Process

The dual nature of South African agriculture (Greenberg, 2017), and of the broiler value chains in particular (Ncube, 2018), has been described in previous literature, and discussed above. The South African case has important specificities, many of which are related to the concentration of land tenure rights in the hands of historically dominant elites, up to and beyond the end of the apartheid regime (Greenberg, 2017). However, the concentration of market share in the hands of an increasingly smaller number of companies is also related to self-reinforcing market consolidation dynamics, which aggravate initial inequalities. This self-reinforcing process is depicted, in a simplified form, in Panel 1, Figure 1. These dynamics are not exclusive to South Africa, and have been described in other contexts and at a global scale: Market consolidation processes tend to reinforce themselves through economies of scale (Sexton, 2000) (which lead to larger companies being more efficient), unequal propensities to invest, and investment returns (Covarrubias et al., 2019) (where larger companies invest more, with higher benefits).This is reflected in loop (R1a) in Figures 1, 2. Other reinforcement processes are not related to increased efficiency of larger companies, but rather to the accumulation of market and political power, which lead to increased barriers to entry for smaller producers (McCorriston, 2002; Greenberg, 2017)and corporate capture of regulators (Mindell et al., 2012) (See loop R1b, Figure 2). The two linked feedback loops shown in the figure lead to what is known as a “success to the successful” archetypal behaviour (Wolstenholme, 2003). The role of policy intervention, in this context, is to counteract this reinforcing dynamic, attempting to lead the system towards a less polarised state. Although the focus of this study is on small producers rather than integrated producers, both segments are heavily inter-linked in terms of their supply of day-old-chicks, feed, veterinary services and more and these linkages are crucial to understanding the challenges faced by small producers.

### Reliable Access to Affordable Inputs and Credit

Our stakeholder interviews confirmed existing literature, identifying restrictions in input access as an important challenge for medium and smallholders (Louw et al., 2017). In the following section, we unpack some key underlying mechanisms and feedbacks underpinning input access restrictions. Partial CLD are shown on Figure 1, panels 2 and 3. The feedback loops are also shown in the final CLD on Figure 2

### Restricted Access to Key Inputs and Excess Capacity

An important barrier for emerging producers is the issue of restricted availability of day-old chicks. This was specifically identified as a barrier for independent producers wanting to scale up beyond a certain size and compete in the formal market with established integrators. Interviewees from different backgrounds focused on the restricted availability of parent stock and the exclusive franchise for the genetics of the two main commercial breeds of broiler (Ross from Aviagen and Cobb from Cobb-Ventress), which, in South Africa, are each owned by one of the two largest integrated broiler producers. Alternative commercial breeds are not widely available.

One interviewee, involved in providing extension veterinary services and training to small and medium producers, described how restricted availability of inputs combined with a lack of awareness of these difficulties to frustrate the expansion plans of emerging farmers, and how the bottle-neck can be traced back to parent stock:

He thinks well if I can produce day-old chicks, I would become rich. Because there is a shortage. But he did not realise what was the complete business plan or impact of this. So, then he would say I want hatching eggs. I have got the machine, but I have not got hatching eggs. So, I said you are not going to get hatching eggs, because there is a shortage of hatching eggs. That is why you have got a shortage of day-old chicks. So, he said okay, now I am going to produce my own eggs. And I said okay you can produce it, but you need parent stock to do that. And you need at least four parent stocks […] Interview code (IC) 190515_002. Support supply chain. Service providers

The restricted availability of breeding inputs is also framed as a symptom of independent producers being “locked out” of the mainstream supply chain, as conveyed by another interviewee:

It got to a point where we could not get the day-old chicks as and when you wanted to get them, which became a problem, because if you can’t get your production [inputs], then you can’t produce. […] It’s not that they’re not available, they’re not available for you. […] And when you are growing 200, 300, 500, you get them with no problem, but the moment you want big numbers, you don’t get. IC 190613_001.Broiler grower, mixed live and slaughtered sales

Policy makers identified the current franchising system as a challenge for small and emerging producers, pointing out that the broiler sector […] works like Microsoft. You buy your computer but you still have to buy software. IC 190624_002. Government official

A parallel State franchise, with a smaller competitor of Aviagen and Cobb-Ventress, was suggested by one interviewee as a potential solution to the issue, reinforcing the narrative of independent producers being locked out of corporate supply chains for breeding inputs. Even if farmers still had to pay, and although it would not shelter them from competition with current franchise-holders, it was suggested that a parallel franchise might significantly contribute to eliminating the existing bottle-neck:

[…] If the State would then have its own centres – it’s not competing with these other centres, because already the franchise for South Africa has been gotten by someone but if you’ve got additional parent stock franchise in the form of the State. These people will not get… farmers will not get it for free, they will pay, to enable the State to service that franchise– Then there will be a constant and frequent flow ofchicks to the farmers to grow. IC 190624_002. Government official

Public subsidies for farm infrastructure (CASP, 2014), combined with unaddressed restrictions in input access, contribute to creating excess capacity. Excess capacity is generally associated to reduced efficiency, increasing average and marginal costs, as fixed costs are distributed across fewer units.

The people that were funding us, then they did not fund us fully […] They funded us for the acquisition of the farm, but they did not fund us for the establishment of the breeder flock. […] So, at this stage we are producing, but we are still buying eggs, and we are in the same cycle that we were in where you don’t get them when you want to […] For example, that side we can do about 15 000 chicks a week. […] the maximum we went to was 8000. We couldn’t go bigger. We’ve never done 10 000, but the space is there. We even have got the abattoir that one can use. It’s not running, because there is no product to slaughter continuously. IC 190613_001

The loop structure in Panel 2, Figure 1 shows the dynamics described above by interviewees. The small market share held by small and emerging producers prompts policy actions to support them, in this case through subsidies for farm setup and infrastructure provided as part of national agricultural development initiatives (DAFF, 2017b). These are meant to increase efficiency and market share in the sector (balancing loop B2a). The availability of public funds creates expectations of market opportunities and leads to an increase in small producers installed capacity. However, restricted access to breeding inputs (eggs, day-old-chicks, parent and grandparent stock), create a bottleneck, and lead to excess capacity, which reduces efficiency, given that fixed costs need to be distributed across a larger number of units (reinforcing loop R2a).

### Transaction Costs, Liquidity Gaps and Coping Strategies

Feed represents around 60% of the input cost for broiler growers. Unlike the breeding stock, feed is not under an exclusive national patent for each breed. Although the feed market is dominated by integrators, there are some large independent competitors who sell primarily to independent medium or small producers. There is not, therefore, a bottleneck for feed affecting small and emerging producers of the type discussed in Restricted Access to Key Inputs and Excess Capacity Section for breeding inputs.

However, our research corroborates previous literature, which has pointed towards high transportation costs and lack of bulkbuying as important factors driving up feed prices for small producers (Louw et al., 2017). In this section we discuss how, in the absence of liquidity and credit, farmers have no choice but to resort to coping strategies which can ultimately aggravate the issue of high transaction costs, undermine efficiency, and perpetuate existing challenges.

The members of a small cooperative mentioned, for example, complementing commercial feed with maize (mielies) to cut cost.

I give them the commercial feed, for the period of four weeks. Then after four weeks I start mixing the commercial feed and the mielies (maize). After that, when they grow big, I put out the mielies (maize) and then they can pick the mielies. […] The reason for this is saving the cost and cutting the chemical. IC 190606_002. Broiler grower. Women’s cooperative, live sales

Commercial broiler breeds, which have been genetically selected for fast growth and efficient feed conversion, require commercially prepared feed in order to realise this genetic potential. Broiler chickens do not fare well if left to scavenge or if fed a non-optimised feed mix, which varies throughout the production cycle (Alqaisi et al., 2017). Mixing commercial feed with maize, therefore, as described above, might cut costs in the short term but is likely to considerably undermine technical efficiency.

A representative from a different cooperative referred to splitting planned feed purchases across several trips to manage cash-flow following an unforeseen price increase:

[Referring to frequent changes in feed prices] They affect me a lot because when I have an amount of money, and I go there to get fifteen bags and the cost has changed, I have to go down to twelve bags, or ten and I have to go there again. You know what I mean? Afterwards, so that I can reach the amount of chicken I have. IC 190531_004. Broiler grower. Men’s cooperative. Live sales

Given that many farmers have to travel considerable distances to obtain feed, if credit restrictions lead to additional, potentially unplanned additional trips, this can add substantially to transaction costs.

Members from a large feed production company selling directly to smallholders identified the issue as being widespread, explaining the decision to market smaller feed bags to address the combination of high prices and credit restrictions:

We have also done 10kg bags and we will short[ly] be doing 20kg bags again just for the people who do smaller numbers or that don’t have the cash in hand to pay for 40kgs but buy 10kgs every few days as you have money coming in. IC 190618_001

Aggregation of input demand was discussed as a partial solution, as it could allow smallholders to bypass local retailers who keep an important margin on the feed and deal directly with feed companies, as well as saving on transport costs, ultimately “*getting bigger loads into [rural] areas and shared loads.” IC 190618_001*

Although an analysis of the price fluctuations of maize and soy feed inputs is beyond the scope of this study, it is worth pointing out the potentially important role of not only local feed markets but also more distal factors, such as drought and dynamics in international grain commodity markets.

Besides the issues discussed above, our research suggested other factors which can restrict access to high-quality inputs. In particular, some interviewees mentioned difficulties in accessing affordable antibiotics, drinking water for the birds, and reliable electricity or gas for heating. We do not have enough information for a more in-depth analysis of every one of these factors, which might be highly context specific. However, such issues can also create important challenges and should be considered in the context of any intervention aiming to support smallholders.

The dynamics described in this section are represented in the causal loop diagram Panel 3, Figure 1. Small producers face liquidity gaps when available cash is not enough to cover operational costs, particularly feed costs. They then engage in various coping mechanisms (smaller and more frequent purchases of feed, mixing specialised feed with maize), which reduce short-term operational costs (B3a) but undermine longer-term efficiency, increasing transaction costs, and reducing output (R3a). Together these loops form a dynamic which is similar to a common system dynamics archetype, known as a “fix that fails” (Wolstenholme, 2003).

### Market Access

Another important challenge discussed in previous literature, and corroborated by our findings, is the lack of a reliable market for smallholders’ output (Louw et al., 2017; von Loeper et al., 2018). Please see Figure 1, panel 3 for the partial CLD, and the full CLD in Figure 2.

Selling small to medium batches into the channels currently available for formal commercialisation is widely considered unfeasible (Louw et al., 2017), as large integrators and supermarkets are not considered to have incentives to include small independent producers into their supply chains. An interviewee who had experience in veterinary service provision to smallholders explained:

We had an approach from a person who has got a 10.000 bird farm and he just said, I have no idea where we would slaughter those. There is no one, you are not going to build that into any company’s system. IC 190520_001. Support supply chain. Service providers.

Parallel marketing systems are discussed as a potential component of a solution. These can include aggregation schemes, where a cooperative focussing on live sales which would preserve the premium that farmers obtain in the live sales market or perhaps an independent abattoir, for example, is supplied by several smallholders in a coordinated fashion. However, interviewees highlighted some challenges in this regard as well.

Lack of certainty in being able to place the full batch, as well as cash flow issues, which were also discussed in the previous section in relation to input purchases, combine to perpetuate multi-generational production systems. Multi-generational operations, as opposed to an all-in all-out approach, can undermine the biological performance of birds, making it harder to optimise the environment, and increasing the risk of disease transmission across generations (Butcher, 2002; Sharif et al., 2014). These issues are in addition to the transaction costs involved in procuring age-specific medicines and feed inputs for birds. A cooperative member described his attempts to maintain an all-in all-out system

When they are six or seven weeks, I [try to] slaughter them and sell them in a group at one time; so that I can get the next one quickly. [… but…] Then about five are left and I can’t mix them with the others. IC 190531_004. Broiler grower. Men’s cooperative. Live sales

Other interviewees, providers of feed for smallholders, emphasised the feedback loop created when credit restrictions lock farmers into multi-generational production systems:

Obviously your multi-aged system, if you are a one-man-band you can’t afford to place everything and wait for 5 or 6 weeks and sell everything. […] So there are still [small producers] wanting to place every week and needing to place every week because they need that steady income to buy feed for the next batch and the whole thing needs to roll. IC 190618_001.

### Market Aggregation Mechanisms

For this sub-section see panel 4 in Figures 1 and 2 for full CLD.

Market aggregation mechanisms, especially those that focus on live sales, preserving the price advantage small producers get by targeting this market segment (DAFF, 2012; Louw et al., 2017), can help support small and independent producers.

Attempts to create market aggregation systems without addressing the issue of cash flow for smallholders, however, are likely to fail, as producers might have limited incentives to fully participate, which can ultimately undermine the viability of the aggregation system. In the words of one interviewee:

The temptation [is] to sell birds over the gate, put money in their pocket for cash, over-state the mortality on their records and then [the abattoir/aggregator] sits with 90 000 birds instead of 100 000 because there is a gap.” IC 190618_001.

The dynamics described in this section are shown in the feedback loop diagram Panel 4, Figure 1. The existence of a liquidity gap, and the uncertainty of being able to place the entire batch, create incentives to sell small numbers of live birds mid-way through the cycle, in the informal market (B4a). This strategy locks producers into multi-generational systems of production which, as discussed above, are associated with reduced efficiency (R4b), and makes it harder to establish market aggregation mechanisms. In the event that an aggregation mechanism had been put in place, additionally, this coping strategy can undermine the financial viability of the aggregator, leading to eventual failure (R4a), reinforcing the above-described lockin. There are various mechanisms through which on-the-side sales and over-reporting of mortality can lead to failure of the aggregation system, including through losses associated with compensation payments, or through unfulfilled orders and client loss. Loop R3a is generic and does not specify any particular mechanism. Loops R4a, B4a and R4b create a dynamic similar to the system dynamics archetype known as “accidental adversaries” (Wolstenholme, 2003), where the strategies of actor A (in this case, any small producer) involuntarily undermine actor B on whose performance or continued existence actor A’s success ultimately depends. Finally, the increased risk associated with multi-generation operations selling in unpredictable informal markets can reduce their access to credit, creating an additional reinforcing loop (R4c).

### Knowledge, Information and Skills

Lack of business and technical knowledge has been emphasised as a challenge for smallholders both in the literature (Wynne and Lyne, 2003) and by study participants. Business skills, it was emphasised by experts and service providers working with small producers, could reduce excess capacity by aligning expectations with actual market shares for small producers. The need for the technical skills was emphasised by producers, but was not identified as a stand-alone solution, and material challenges were referred to as more important.

One informal cooperative member also referred to barriers to accessing help, related to time, and perhaps difficulty in elaborating the required documentation or perceived difficulty of accessing support schemes:

If the government want to help us with chicks, we would accept that, but we have never asked for assistance and we don’t have time to write business plans because we are busyIC 190606_002

Producers spoke of useful training and knowledge transmission but contrasted this with a lack of real opportunity or real “help”. One said:

They send us a WhatsApp or email of some sort to come to this meeting, to [nearby town] or whenever there is a thing going on. So, we always learn more about the chickens. […] But they always say-keep on pushing guys! And we keep on pushing, and we’re pushing and pushing and pushing. And they never help us IC 190531_004. Broiler grower. Men’s cooperative. Live sales

Overall, the lack of knowledge and skills could perhaps be considered a cross-cutting issue which aggravates other challenges, rather than as a separate challenge itself.

### Policy Entry Points, Complementarity and Coherence

#### Policy Entry Points

Policy areas potentially affecting the broiler sector include agricultural development policy, environmental impact assessment, foreign trade policy, public health and food safety standards, food and nutrition security, and private corporate governance of leading companies (integrated producers, retailers and quick service restaurant segments or fast food). Both the thematic analysis of interviews and the document analysis identify agricultural development policies as the most relevant for small broiler producers. These policies mainly involve the former Department for Agriculture Forestry and Fisheries (DAFF), as well as the Department of Rural Development and Land Reform (DRDLR) of the Department of Trade and local implementing authorities. The agriculture functions of the former DAFF are now incorporated now incorporated into the Department of Agriculture, Land Reform and Rural Development (DALRRD) The Department of Small Business Development (DSBD), created in 2014, also has responsibilities over small farmer promotion.

Based on the analysis of interviews and policy documents, key entry points for agricultural development policy have been identified, which address the challenges described in the above section.

These entry points marked in red in the full causal loop diagram (Figure 2), and listed in Table 2 include grants or other policies to support input access and infrastructure, credit provision, training, and aggregation schemes, both for input purchase and market access. In the diagram, training is split into business training which can improve efficiency but also support better realistic assessment of market opportunities, and technical training directly addressing producer technical efficiency. These entry points for policy would act by reducing transaction costs and increasing bargaining power in order to facilitate input access They would also target input bottlenecks associated to current structures in the large-scale commercial segment. Finally, by facilitating credit, liquidity, training and aggregation mechanisms, they can support efficiency and sustainable improvements in market access.

#### Complementarity and Coherence Across Key Policy Entry Points

In general, complementarity results when policies are implemented that increase the efficiency of another policy (Howlett and Rayner, 2007), address its negative externalities, or target a different driver of a common goal. Coherence arises when policy interventions systematically seek complementarity and eliminate conflict (Den Hertog and Stross, 2011; Nilsson et al., 2012).

Based on the analysis of policy documents and interviews, policies are mapped onto the CLD, and their main targets, and potential constraints are identified.

The resulting full CLD highlights the complementary nature of the key policy entry points identified. Firstly, key policies target different challenges which ultimately impact the common goal of supporting small producers. Secondly, interventions enhance each other’s efficiency by removing constraints to their effectiveness described in causal loops R1a to R4a, which configure commonly-known archetypes explaining systems malfunction (see Table 2). For example, in the absence of credit, investment in aggregation mechanisms might fail to achieve its objectives, if farmers’ short-term coping strategies undermine their participation and their efficiency. Likewise, the effectiveness of input credit would be undermined in the absence of improved market access. The analysis suggests that effective intervention to support small producers would benefit from internally coherent agricultural development policy addressing these key points. Implementation would require the participation not only of small producers but of actors downstream, upstream and in the support supply chain. However, key interventions identified do not necessarily rely on modifying the behaviour of current mainstream actors (either integrators acting upstream or supermarkets acting downstream) but could rely on setting up alternatives to mainstream value chains. These could complement other regulatory efforts aimed at leading companies upstream and downstream from small producers (Aliber et al., 2013).

#### Policy Coherence in the Current South African Policy Environment

Both the interview analysis and the review of policy documents have been used to contextualise our analysis within the South African policy environment.

All of the entry points identified in the study have been targeted by different policies at different points in time (see Table 2). In particular, the Comprehensive Agricultural Support Programme (CASP, 2014) has focussed largely on providing grants for farm set-up and infrastructure, while MAFISA is a programme aimed at improving access to credit (DAFF, 2019). Several initiatives have attempted, in different regions, to invest in value chain integration and set up abattoirs to improve farmers’ access to markets, including the Agri-Parks initiative (DRDLR, 2016). There are also several programs aimed at providing training to smallholder farmers in technical and entrepreneurial skills (CASP, 2014; AgriSETA, 2018).

Interviewees from various backgrounds acknowledged the government’s efforts to support small and emerging producers. However, participants in our study generally perceived current intervention as being insufficient hard to access, fragmented and inefficient two interviewees summed up this perception:

Yes, there will be incubators, there will be everything and the problem is that when things are funded, no one comes and talks to people in the know like this. We have got R1 000 000.00, we think an abattoir would be a good idea so let’s put it there, spend the R1 000 000.00, take it off the bottom line, get your BEE [Black Economic Empowerment] points, all good, wash your hands of it and then you have got a white elephant. That one at [Location], the water had never been connected to that abattoir, everything was in there… IC190618_001/Participant 1. Feed suppliers

He has got a chicken run, you will find him and then as I said there is no follow up. Nobody is coming to say, the government gave him so much money, is he doing it right, you know that is the sad part. 190618_001/Participant 2. Feed suppliers

Publicly available parliamentary committee progress reports also point out the fragmented nature of interventions. Insufficient funding has led to the abandonment of fundamental pillars of existing intervention programs (DLRRD, 2019), moving away from what was initially conceived of as a more systemic approach. Other interventions were perceived as competing with each other, such as MAFISA credits and CASP grants, instead of acting in a complementary way, targeting different entry points (e.g., Capacity installation versus operational costs) (DAFF, 2019). Other more unconventional policies suggested by participants, such as the establishment of a parallel state franchise for commercial breeds, have not been carried out.

Policy progress reports (DLRRD, 2019), and recent initiatives such as the efforts to put in place a Poultry Masterplan (Poultry Masterplan, 2019), and the recent creation of DALRRD, merging land and rural development with the agriculture competencies of former DAFF evidence a growing recognition and political will for systemic agrarian transformation, despite remaining challenges (National Assembly, 2021).

## Discussion

### Implications for Local Stakeholders and Policy-Makers

This study highlights the importance of internally coherent agricultural development policy in South African food systems. A transformative policy agenda informed by the present analysis would target key leverage points in the system (addressing input and market access, liquidity and credit) through complementary policy interventions, with the aim of interrupting the selfreinforcing dynamics that exacerbate duality in the value chain (including accumulation of market and political power, underutilised capacity, efficiency-eroding short-term coping mechanisms and the inability to move away from multi-generational broiler production systems). The resulting focus on strategic complementary intervention across key areas would be clearly distinct from “silver bullet” policy recommendations, without falling either into the category of “holistic” approaches, where it is assumed that “all types of support must be provided at once” (Aliber et al., 2010).

The coherence of agricultural and food policy in South Africa is recognised as a wider challenge, and has been identified as lacking in the contexts of national nutrition security policy (Thow et al., 2018), food safety in the retail sector (Boatemaa et al., 2019) and nutrition-sensitive agriculture (Schönfeldt et al., 2017). There have also been several attempts to adopt coordinated system-wide policy agricultural development policies which have, nevertheless, not been fully and adequately implemented (DAFF, 2015, 2019).

The findings from the present study are consistent with previous research on the topic which analyses the role of liquidity constraints as a challenge for South African smallholders (von Loeper et al., 2016) and analyses challenges for independent broiler producers in the region including lack of market access and insufficient knowledge (Wynne and Lyne, 2003; Louw et al., 2017). Our findings add to the literature on this topic, illustrating the interlinked and reinforcing nature of the mechanisms that underpin value chain duality and smallholder disadvantage and situating potential interventions within the broader South African policy landscape. Additionally, as is often the case in complex systems analysis, many small producers’ behaviours that appear to be dis-functional (certain coping mechanisms, underutilised capacity) emerge as rational within context, with small producers acting as “locally constructive agents” (Sinitskaya and Tesfatsion, 2015), which would suggest the need to address wider systemic incentives rather than directly targeting said behaviours. That implies that, while training is undoubtedly an essential component of smallholder support programmes, it would not, by itself, solve the challenges faced by small producers.

The set of constraints identified could be, in principle, addressed by a number of different policies or initiatives. This could include a combination of policy elements familiar in the South African agricultural policy environment, such as grants, loans, credit guarantees and the creation of cooperatives or other aggregation mechanisms to facilitate market and input access constraints, as well as training and mentoring programmes.

In terms of government capabilities, a policy package coherently addressing existing constraints would probably rely strongly in the areas of information management, organisational capacity and financial resources, while relying less on governmental “authority” (Hood, 1987).

This type of approach could potentially increase smallholder market shares, improving performance and income stability. The inherent unit cost disadvantages related to scale would most likely remain (Louw et al., 2017), implying that integration of small producers within the mainstream supply chain is unlikely, and smallholder promotion initiatives would most effectively target the creation and development of parallel avenues for marketing.

Changes in discourse, attitudes and causal beliefs are recognised as fundamental components of transformational change (Polanyi, 2001). However, the realisation of transformational impact is likely to depend on to what extent and how the insights from the present systems analysis are used by small producers and advocates to challenge defeatist narratives and discourses that focus primarily on smallholder inadequacies. An informed systems perspective might be particularly useful, for example, in the context of high-stakes policy processes involving negotiation between government and integrated producers (Poultry Masterplan, 2019) and where there are opportunities for small producer advocates to exert influence.

### Methodological Insights

The above section has discussed the potential for complex systems approaches to provide relevant insights in the context of smallholder promotion and inclusive food systems. This includes identifying interlinked challenges, reinforcing dynamics and policy levers, as well as challenging simplistic narratives and interpretations and causal assumptions, and providing alternative discourses.

However, there are also some important challenges. Like many policy areas in agricultural development and food systems, the issue of smallholder promotion, particularly in industrialised and dualistic food value chains, is characterised by the interaction and confluence of conflicting interests (Losch, 2004). These are negotiated through complex political processes, involving stakeholders who differ not only in terms of perspective but also of goals, knowledge culture and power within the system (Leach, 2008).

Systems analysis methods, on the other hand, often place a strong emphasis on building consensus around proposed solutions, often through participatory group model-building approaches (Winz et al., 2009; Rich et al., 2015). While the inclusion of diverse perspectives is considered central, conflict of interest is acknowledged only to an extent, and often within the assumption that, although different groups might benefit to different degrees from a proposed policy, it should be possible to overcome such relative conflicts of interest as part of the system analysis process (Rich et al., 2015). Adopting a different framing, Rammelt and Leung (2017) argue for the use of systems approaches to analyse solutions that appear to be a win-win, while in fact producing winners and losers. However, negotiated outcomes, in highly contested policy areas are likely to require compromise rather than consensus. The actual transformational impact of systems analysis research carried out by external, non-commissioned researchers is then likely to depend on the use that different stakeholders make of systems-informed narratives in the context of wider policy processes.

Group model building has been shown to be highly constructive and to offer many advantages (Lie et al., 2017; Totin et al., 2018). However, the associated framings discussed above can constrain the use of systems analysis to contexts where there is the potential for a win-win or consensus-achieving solution, and where researchers are in a position to lead a consensus-building process. An exclusive focus on “consensus building” approaches also risks not fully engaging with issues of conflict of interest and complex policy processes which are as much part of the food system as more logistical aspects of supply chains.

In order to maximise the usefulness, applicability and transformational potential of systems approaches to promote inclusive food systems, would recommend careful consideration of the wider policy context within which research is taking place. The adoption of critical systems thinking as proposed by Smith (2011), and as adopted in this study is helpful. Additionally, we recommend that trade-offs between different methods of individual and group-based stakeholder participation are considered in relation to conflict of interest and pre-existing power dynamics. The most context-appropriate method or combination of methods should be chosen to ensure that relevant conflicts of interest are captured and participation of all stakeholder groups is supported. We would also suggest that food systems analysis is combined with an analysis of the policy context and relevant policy processes. Finally, we suggest the need for further research into the uptake of insights and narratives from systems analysis into wider policy processes, going beyond consensus-building as part of the research process itself.

### Limitations and Further Research

The present study has some limitations which should be considered when interpreting the results.

Firstly, this is an entirely qualitative systems analysis and, as such, it does not allow for quantitative prediction, policy evaluation or formal scenario simulation.

Secondly, this analysis is based on individual and small-group interviews, as opposed to group model building (Van Kerkhoff and Lebel, 2006). This methodology was deemed appropriate given the context, the nature of stakeholder relationships and the positioning of the team itself as external, non-commissioned researchers, all of which have been extensively discussed earlier in this section. While the methodology adopted offered advantages in terms of guaranteeing inclusive stakeholder participation in our context, it can limit transformative outcomes through stakeholder networking which have been found to be relevant in other contexts (Totin et al., 2018).

The influence and use of insights from systems analysis in wider policy processes has been identified as a priority area for further research. Quantitative scenario simulation and evaluation of specific policy designs addressing the key system leverage points identified are also potential areas for further research in the context of our study.

## Conclusion

This study has developed a thematic and qualitative CLD analysis, focussing on the challenges faced by small and emerging producers in the South African broiler sector and potential interventions to address them.

The main challenges include a lack of reliable access to day-old-chicks for producers wanting to scale up (a “bottleneck”), high costs for other key inputs such as feed, resulting from high transaction costs and lack of bargaining power, and limited access to output markets, with producers being unable to reliably place their batches at the end of a cycle. These challenges are compounded by a lack of liquidity and the consequent reliance on short-term coping strategies, as well as producers’ insufficient technical and commercial skills.

The above challenges can exacerbate each other and lead to self-reinforcing dynamics mediated by the accumulation of market and political power, the creation of overcapacity in the sector, efficiency erosion and the failure of attempts to implement aggregation mechanisms or to shift away from less efficient multi-generational production, towards a staggered, all-in all-out systems.

The analysis suggests that a coordinated intervention package, targeting key leverage points, could disrupt these dynamics and promote a shift towards a more inclusive value chain, supporting smallholder livelihoods and increasing their market share. Key entry points include: Firstly, direct support for input access and improved infrastructure via grants, subsidies or other policies; secondly, provision of credit and liquidity for operational expenses; thirdly, investment in in input and output market aggregation mechanisms and parallel marketing channels fourth, regulations or initiatives directly addressing structural barriers in the commercial segment in order to facilitate access to day-old-chicks; finally, holistic training in both business and technical skills.

Together, these policies would contribute to reducing transaction costs, improving bargaining power and promoting efficiency, all of which could support livelihoods and improve market shares.

It is beyond the scope of this study to recommend or evaluate specific policies. However, many of the interventions which have been already implemented in the South African context could be effectively combined to target the above leverage points, attending to their linkages and interactions. This could include grants schemes, credits schemes, liquidity guarantees and training programmes, as well as the creation of cooperatives, hubs or other aggregation and marketing schemes.

Dealing with conflict of interest and contested policy processes are identified as challenges for systems analysis methods in the area of smallholder promotion and inclusive food systems, but also as areas where there are substantial contributions to be made.

Recommendations for future systems research in this area include careful engagement with and analysis of issues of conflict of interest and ongoing policy processes. This can inform deliberate choice and combination of stakeholder participation methodologies appropriate to such contextual issues. Further research is needed to better understand how narratives and solutions emerging from complex systems analysis influence policy processes in food systems.

## Figures and Tables

**Table 1:** Analytical steps: identification of challenges, key variables, cause-effect mechanisms and feedback loops in the system.

Analytical step	Description
Thematic analysis	Full thematic analysis of supply chain stakeholder interviews and extraction of key quotes relating to challenges faced by small and emerging producers and relevant policy entry points. Analysis of policy documents to triangulate with interviews.
Cause-effect analysis	Cause-effect analysis of key quotes from interviews and policy documents, identifying relevant variables and causal links. Based on Kim and Andersen (2012).
Coding of variables and generalisation	Codes are assigned defining more general variables. This analytical step is also based on the methodology described in Kim and Andersen (2012).
Identification of feedback loops and archetypal structures	Feedback loops emerging from the generalised cause-effect analysis are identified, as well as archetypal system dynamics structures which are formed by two or more feedback loops Wolstenholme (2003).
Graphical representation (Causal Loop Diagram)	Graphical representation of the resulting causal structure in the form of partial causal loop diagrams (Figure 1, panels 1 -4), which are integrated into a full diagram (Figure 2).
Policy implications	The findings from the thematic analysis and resulting CLD are interpreted in terms of their implications for policy-makers and stakeholders and compared to recent policy practises in South Africa.

**Table 2:** Causal loop diagram elements: feedback loop codes, descriptive naming, loop sign and correspondence to archetypal system dynamics structures.

N	Feedback loop/Node labels	Feedback loop/Node name	Sign	Archetypal structures
1	R1a	Duality reinforces market dominance of integrators	Reinforcing	1 and 2 Success to the successful
2	R1b	Duality reinforces disadvantage of SEP	Reinforcing	
3	R2a	Pro-SEP policy support aims	Balancing	3 and 4 Fix that fails
4	B2a	Overcapacity	Reinforcing	
5	B3a	Short-term cost reduction	Balancing	5 and 6 and 7 Fix that fails
6	R3a	Coping mechanisms: Liquidity gap trap	Reinforcing	
7		Multi-generational production	Reinforcing	Not explicitly shown in CLD. Would simply reinforce the dynamics in 5-7
8	R4a	Market aggregation failure	Balancing	8 and 5 Accidental adversaries
		**Entry points**	**Main correspondent Policy interventions**	
9	[p1]	Subsidies for farm set-up and infrastructure	Comprehensive Agricultural Support Programme (CASP), others*	
10	[p1b]	Input packages: Provision of essential inputs for starters	Comprehensive Agricultural Support Programme (CASP), Ilima/Letsema, others*	
11	[p2a]/[p2b]	Training	AgriSETA, others. This component is theoretically included in CASP but under-funded	
12	[p3]	Credit for operational expenses	MAFISA: Discontinued for lack of funds, perceived as competing with CASP, others*	
13	[p4]	Investment in aggregation mechanisms, value chain upgrading	Agri Parks scheme. Only partially implemented due to lack of funds. Perceived failure to coordinate with local actors and complement other policies. Others*	
14	[p5]	Parallel state franchise for broiler breeds	Proposed by participants, not implemented	

Others*: New schemes under the National Policy for Comprehensive Producer Development Support and SMME Support Plan. These have only been recently implemented and there is limited information about their effectiveness and implementation.

## Data Availability

The data used for this study can be made available, in an anonymized form, upon request from the authors. Full interview transcripts can not be shared to preserve anonymity.
